# On Hernia e diverticulo Intestini (Hernia Litrica), with Remarks on Fæcal Fistula and Artificial Anus

**Published:** 1842-10

**Authors:** 


### ART. II.

*Ueber Darm-Anhangs-Brüche (Herniæ Litricæ). Mit Bemerkungen über Kothfisteln und Widernatürlichen After.* Von Dr. C. F. RIECKE.—*Berlin*, 1841. 8vo, pp. 192.

*On Hernia e diverticulo Intestini (Hernia Litrica), with Remarks on Fæcal Fistula and Artificial Anus.* By Dr. C. F. RIECKE.—*Berlin*, 1841.

FEW diseases are of greater interest and importance than herniæ of the intestinal canal, and few, if any, have been more ably and successfully investigated; their pathological anatomy especially has been so minutely and diligently examined that we might almost suppose this department of the subject was exhausted. Dr. Riecke, however, in the treatise which forms the object of this notice, directs attention to a variety of hernia hitherto overlooked, though, according to him, it is of frequent occurrence and presents peculiarities of importance in a practical point of view.

In the *Mémoires de l'Académie des Sciences* for the year 1700, LITRE describes two cases of inguinal hernia in which the proper caliber of the intestinal canal was not included in the hernial sac, the protruded portion of intestine, consisting of a digital prolongation of the ilium, which Litre concluded was formed by the gradual extension of a knuckle of the bowel that had become engaged in the inguinal canal. Litre's observations have, on the whole, attracted little attention, and been inaccurately understood. It appears obvious that his cases were examples of hernia of a diverticulum of the intestine, and to such cases should the appellation *hernia Litrica*, adopted by some authors, be confined. The term, however, has been so loosely applied that its real import has been usually quite lost sight of. Thus, for example, Rust, in his *Handbuch der Chirurgie*, comprises under the names *hernia lateralis* and *hernia Litrica* every small hernia, in which but a portion of the caliber of a hollow viscus, whether stomach, intestine, or urinary bladder, is contained in the hernial sac. To avoid such confusion of terms Dr. Riecke designates the *hernia Litrica* by the name *hernia e diverticulo intestini*, and calls that form of hernia in which but a portion of the cylinder of the intestine is prolapsed *enterocele partialis*. Having premised thus much we shall lay before our readers a full analysis of Dr. Riecke's work.

The first chapter is little more than a transcript of Meckel's account of diverticula of the intestinal canal, of which it will be recollected there are two kinds. The first is the *diverticulum congenitum*, supposed to originate from the more or less perfect continuance of the connexion of the small intestine with the umbilical vesicle, and in the composition of which all the tunics of the intestine are concerned. The second species is the false diverticulum (diverticulum spurium seu acquisitum) which is formed by a protrusion of the mucous membrane of the intestine through its muscular coat, and therefore consists of its mucous and peritoneal tunics alone.

Both these species of intestinal diverticulum may become engaged in hernia; but the *diverticulum acquisitum* exclusively constitutes the *hernia e diverticulo intestini* of our author. Dr. Riecke enumerates several of the recorded cases in which a *diverticulum congenitum* formed the contents of a hernia, but such cases form a distinct class from those constituting the proper object of his work, and are not further adverted to.

The history of the *hernia e diverticulo intestini* is given in very lengthened detail in the second chapter. According to Dr. Riecke, this description of hernia occurs, perhaps exclusively, in the femoral canal, at least he knows no example of its occupying any other situation. Females are much more liable to it than males, but our author has once met with it in the latter sex. The etiology of the affection is the same as that of hernia generally; the diaphragm and abdominal muscles, especially during any unusual bodily effort, exert a pressure on the abdominal contents, and if that pressure exceeds the resistance offered at any particular point, say the femoral ring, the intestine finds exit, or its muscular fibres separate and allow the mucous membrane to protrude and push before it the peritoneal covering of the bowel; the *diverticulum acquisitum* being thus formed enters the femoral ring, and constitutes the first stage of the *hernia e diverticulo intestini.* The unyielding nature of the boundaries of the femoral ring explains the greater frequency of the affection in that situation; the descent of the intestine *en masse* being opposed, though the passage of the smaller diverticulum is permitted; while the more distensible inguinal ring readily allows the entrance of the entire thickness of the intestine.

The *hernia e diverticulo intestini* is invariably of slow formation; when it has augmented to a certain bulk, to such an extent for example as to constitute an external tumour, it produces changes in the condition of the intestine itself, to which Dr. Riecke attaches much importance, and which he describes with minuteness, not to say prolixity: they may be thus summed up. The descent of the *diverticulum* in the femoral canal necessarily draws the intestine to which it is attached into close contact with the crural ring, and thus alters the line of direction of the bowel, which forms an angle on itself, the apex of the angle being at the point of origin of the diverticulum, and consequently corresponding to the crural ring. This disposition of the intestine (quite similar, we need scarcely say, to what obtains in ordinary intestinal hernia) coupled with the fact, that a portion of its tissues is engaged in the formation of the *diverticulum* causes a diminution of the caliber of its canal at the point whence the *diverticulum* is given off, which diminution of the internal capacity of the intestine Dr. Riecke, to avoid circumlocution, calls the *stricture.* The intestine being thus (especially when the diverticulum has become adherent to the hernial sac,) fixed at the upper opening of the crural canal, the peristaltic motion is interrupted at the fixed point, a circumstance which, together with the existence of the *stricture* and the altered direction of the bowel at the same locality, materially impedes the propulsion of the fæces, causes their accumulation above the stricture, and thus gradually produces a dilatation of the upper portion of the gut, termed by Dr. Riecke, for shortness' sake, the sinus. These several conditions of the parts constantly tend to augment under the prolonged operation of their exciting causes. Below the stricture the capacity of the intestinal canal becomes rather diminished, the more so, the narrower is the stricture, and the greater the dilatation of the *sinus.* The pathological alterations are accompanied by habitual costiveness, liability to colic, and the various and varying dyspeptic symptoms almost constantly experienced by patients labouring under unreduced hernia of any kind or in any situation; but which Dr. Riecke seems to regard as, in some degree at least, peculiar to and characteristic of the particular kind of hernia under consideration. The hernial tumour itself is rarely the seat of any pain or uneasy sensation. When of long standing and of a certain size, the diverticulum communicates with the cavity of the intestine by so narrow a neck, that the liquid and gaseous contents alone of the intestine pass into it, and if any solid material appears to find entrance, its presence suffices to excite inflammation accompanied with symptoms of strangulation.

The *causes and symptoms of strangulation* are next discussed. Dr. Riecke adheres to the phrase *strangulation* as applied to this description of hernia, although he thinks the obstruction is caused by a mechanism very different from that which obtains in other herniæ; inasmuch as in ordinary cases the causes of obstruction exist in the hernia itself or in the rings or canals through which it passes, while in the *hernia e diverticulo intestini* they lie in the stricture and sinus of the intestine.

The most frequent cause of strangulation is an unusual protrusion of the diverticulum, which either causes the descent of the stricture into the crural canal, or merely draws it so firmly against the crural ring that the intestine is flattened and its cavity obliterated. A similar effect may result from the inordinate distension of the diverticulum by gaseous liquid, or solid matters, or, if the diverticulum be adherent to the sac, the same consequences may be produced by inordinate gaseous distension of a coil of intestine lying beneath the neck of the diverticulum and the intestine whence it originates, as the mesentery, stricture and diverticulum may be thus put forcibly on the stretch over the distended intestine. A case is given (pp. 104-10,) as an example of strangulation from this latter cause, but it does not establish the point, as on dissection the diverticulum was found, not engaged in the femoral canal, but loose and floating in the cavity of the abdomen. The entrance into the diverticulum of solid matter, as fæces, or a foreign body, may, it is stated, excite inflammation with symptoms of strangulation; and a case (p. 165) is given as illustrative of such an event, which, it is easy to understand, might readily occur; it is not however superfluous here to observe that the case just alluded to is simply an example of inflammation and suppuration of a femoral hernia which opened as an abscess, and gave exit to a needle, after which recovery rapidly ensued; but there is not a particle of evidence to show that the hernia was of that particular kind, designated by our author as the *hernia e diverticulo intestini.* In one case (p. 90,) where Dr. Riecke found on dissection all the anatomical characters above mentioned as distinctive of the *hernia e diverticulo*, the obstruction and symptoms of strangulation resulted from a partial invagination of the intestine, the *sinus* having sunk into the stricture and stopped it, to use the author's phrase, like a plug.

Dr. Riecke describes the symptoms of strangulation at very great length, but we need only mention those which he considers as peculiar to, or at least as particularly characteristic of, the *hernia e diverticulo*, No pain or uneasy sensation is at first experienced in the hernial tumour, and from six to eight days may elapse before it even attracts the attention of the patient. If however the surgeon detects the existence of the hernia, and practises too frequent and forcible attempts at reduction, the tumour soon becomes tender and inflamed. The pain is at first chiefly referred to the situation of the sinus, which becomes very tender on pressure, and so distended that in thin patients its outline can be felt through the abdominal parietes; general abdominal inflammation and febrile reaction do not set in for several days; throughout the entire course of the affection the progress of the symptoms is unusually slow and attended with marked intermissions which may deceive both the patient and the physician, (pp. 26-37.) If the diverticulum be returned, whether by the taxis or an operation, subsidence of the symptoms of strangulation and evacuation of the bowels follow much more slowly than in other herniæ, often not for many hours or even some days; because the existence of the stricture and the inflamed condition of the sinus of the intestine suffice to keep up the symptoms. If the hernia opens externally the resulting artificial anus usually heals rapidly.

On dissection, the *sinus* is found to be the chief seat of the inflammation, and is, not unfrequently, gangrened and perforated. The intestine above the stricture is greatly distended and inflamed generally; but below the stricture the intestine is contracted, and inflamed to but a small extent; circumstances, we may remark, constantly observed after fatal strangulated hernia, and by no means peculiar, as Dr. Riecke seems to imply, to the form of the affection under consideration. The *diverticulum* itself is inflamed, but less so than the sinus, and is seldom gangrened, unless attempts at reduction by the taxis have been injudiciously persevered in. (p. 31.)

The diagnosis of *hernia e diverticulo intestini* is the next point considered, and, according to the fashion of our author, few things are said in many words. The characters assigned as distinctive are, the gradual formation and smallness of the tumour which, except in cases of very long standing, rarely exceeds the size of a walnut; it being of a roundish shape, slightly elastic, usually smooth, though occasionally somewhat irregular on the surface, and sometimes hard, so as to resemble and have been mistaken for a lymphatic gland; and, finally, if the hernia be reducible, its return is accompanied by a peculiar gurgle rather felt than heard. We need hardly remind our readers that the foregoing statement is but an imperfect summary of the physical characters commonly ascribed to femoral hernia, and obviously cannot be considered as diagnostic of the *hernia e diverticulo intestini.*

As regards the differential diagnosis of the affection when strangulation occurs, the symptoms differ from those of strangulated *enterocele*, in the remarkable slowness of their progress, and in the tumour being exempt from all pain and uneasiness for perhaps several days. Dr. Riecke considers that it may often be confounded with epiplocele, and that frequently there is no certain means of distinguishing it from this latter form of disease, and in this opinion we entirely coincide.

*Prognosis.* Dr. Riecke regards the occurrence of strangulation as less dangerous in this than in other descriptions of hernia, because of the slow course of the symptoms. On the other hand, however, the reduction of the *diverticulum*, whether by an operation or the taxis, may fail to relieve the symptoms, because the *stricture* in the intestine may remain obstructed, and the amount of its contraction can never be determined during life. The performance of an operation Dr. Riecke considers as more likely to be followed by a favorable result than in ordinary enterocele, provided this measure be had recourse to sufficiently early, before the inflammation of the sinus has run too far, and the stricture became too much obstructed by tumefaction of the mucous membrane of the intestine. The slowness of the symptoms, however, admits of hope at a much later period than it could be reasonably entertained in ordinary cases, even up to the 8th, 10th, or 12th day. Gangrene of the *diverticulum*, or its injury by the knife during the operation, is of less consequence than similar lesions of the intestine in enterocele, for the *diverticulum* not being essential to the continuity of the intestinal, an artificial anus consequent on its injury is much more readily cured than one in which the intestine is implicated.

*Treatment.* Dr. Riecke's views respecting the treatment of the affection, whether in the reducible, irreducible, or strangulated state, are pretty much those most generally recognized by modern surgeons as applicable to hernia generally, with the exception indeed of one point, which we shall briefly notice. It has been already stated that the return of the *diverticulum* may not be followed by relief of the symptoms of strangulation, the bowel continuing obstructed in consequence of the existence of the sinus and stricture of the intestine; under such circumstances, Dr. Riecke recommends the administration of metallic quicksilver as the only means of quickly ameliorating this condition of things; this remedy we are told rapidly allays the vomiting and overcomes the obstruction of the bowels; it should be administered in doses of from two to four ounces repeated at short intervals, and the entire quantity given should not exceed two pounds; to facilitate its action and prevent its lodgment in any particular part of the intestinal canal, the patient should move about, or at least frequently change her position in bed. The circumstance above mentioned is not the only indication for the administration of metallic mercury, it being also recommended where the patient absolutely refuses to submit to the operation, when the sac and *diverticulum* are so intimately adherent that the latter can be only partially returned, and when the *diverticulum* from being gangrened cannot be returned and the symptoms of strangulation continue.

The Third chapter, which extends to 20 pages, contains a very diffuse exposition of Dr. Riecke's views respecting artificial anus and fæcal fistula; but we must candidly acknowledge that we cannot very clearly understand his opinions, or perceive their practical importance. Dr. Riecke's object is to show that the hernial sac is that concerned in the formation of the interval (or membranous funnel of Scarpa,) which in artificial anus intervenes between the cutaneous opening and the breach in the intestine. Whether he limits this doctrine to those cases of artificial anus which are the consequence of the destruction of but a small portion of the diameter of the bowel, or extends it to those in which an entire coil of intestine has been destroyed, does not very clearly appear. At page 69 we are told that, "when a hernia of the diverticulum or an enterocele partialis becomes gangrened, the edges of the opening in the bowel become adherent with the hernial sac in the hernial canal, or to the cutaneous opening, and the bowel itself forms the membranous funnel which, according to Scarpa, can only be constituted by the portion of the hernial sac that has escaped gangrene." And again, at page 75, it is said,

"The hernial sac can only contribute to the formation of the funnel communicating with the cutaneous opening, when the intestine has become adherent to the superior orifice of the inguinal or crural canal, and its contents escape through one of these canals clothed by its hernial sac." We must confess our inability to see how these statements establish Dr. Riecke's doctrine or upset that of Scarpa; but as they contain the sum and substance of the author's arguments, we lay them before our readers that they may form their own opinion on the matter.

We have given so very full an analysis of Dr. Riecke's work, that we have left ourselves little room for comment or criticism, which are indeed on that very account the less necessary; but we cannot conclude without a few observations as to the nature and extent of the materials on which the treatise is founded.

From the minuteness of the details and the absolute statements made respecting the pathology and symptoms of the *hernia e diverticulo intestini*, it might be supposed that Dr. Riecke had framed the history of the affection from the observation of an extensive series of cases—but such is not the fact. Upwards of half the work indeed is occupied with an account of 19 cases, but of the entire number four only can be considered as supporting the author's views. Three are cases in which metallic mercury was successfully administered in constipation with symptoms of ileus unconnected with hernia. Four are cases of strangulated femoral hernia, of which two occurred in the author's practice, where an operation was followed by recovery. One is a fatal case of femoral hernia on which the author operated, but no post-mortem examination was allowed. Two are cases of artificial anus, which present nothing that we can discover calculated to support Dr. Riecke's opinions. Four are fatal cases of femoral hernia collected by the author from various sources, in which a post-mortem examination was performed, but in which the anatomical characters assigned to *hernia e diverticulo intestini* are *not* mentioned. All these cases, excepting of course the first three, Dr. Riecke assumes to have been examples of diverticular hernia, because of the gradual formation, smallness, and globular shape of the tumour, and the slow progress of the symptoms of strangulation. Four of the cases, however, the 1st, 2d, 3d, and 6th, are examples of strangulated femoral hernia which terminated fatally, and were examined after death by Dr. Riecke, and they entirely correspond to his account of the affection under consideration. These four cases in fact constitute the whole foundation on which the work is written; and though they certainly establish the existence of the peculiar form of hernia to which our author has directed attention, they obviously do not warrant the positive and dogmatic history which is given of it. We have nevertheless thought it right to put our readers fully in possession of the contents of Dr. Riecke's work, inasmuch as we believe he is the first who has noticed the peculiar form of hernia which he describes. Several authors, as is well known, mention having found a true diverticulum forming the contents of a hernial sac, but we are not aware of a single instance in which a *diverticulum spurium* has been observed to constitute a hernia. Cruveilhier, for example, (*Anatomie Descriptive*, t. ii. p. 492,) in describing the two kinds of diverticula of the intestinal canal, says that he has on several occasions seen the true diverticulum engaged in hernia, but makes no mention of having observed a similar fact with regard to the false diverticulum.

